# Childhood obesity directly increases age-related macular degeneration risk: the role of physiobiological and immune-metabolic function

**DOI:** 10.7189/jogh.15.04164

**Published:** 2025-06-27

**Authors:** Xinyu Zhang, Yikeng Huang, Mingming Ma, Chuandi Zhou, Yujin Jiang, Zixuan Zhang, Xinyu Zhu, Chenxin Li, Xun Xu, Ying Fan, Changjing Han, Zhi Zheng, Shuzhi Zhao

**Affiliations:** 1 Department of Ophthalmology, Shanghai General Hospital, Shanghai Jiao Tong University School of Medicine, Shanghai, China; 2 National Clinical Research Centre for Eye Diseases, Shanghai, China; 3 Shanghai Key Laboratory of Ocular Fundus Diseases, Shanghai, China; 4 Shanghai Engineering Centre for Visual Science and Photomedicine, Shanghai, China; 5 Shanghai Engineering Centre for Precise Diagnosis and Treatment of Eye Diseases, Shanghai, China; 6 Department of Ophthalmology, Shanghai Key Laboratory of Orbital Diseases and Ocular Oncology, Shanghai Ninth People's Hospital, Shanghai JiaoTong University School of Medicine, Shanghai, China; 7 Department of Ophthalmology, The Second Affiliated Hospital of Xi’an Jiao Tong University, Shaanxi, China; 8 Shengli Clinical College of Fujian Medical University, Fuzhou, Fujian, China; 9 Ningde Municipal Hospital, Ningde Normal University, Fuzhou, Fuijan, China; 10 Fujian Medical University, Fuzhou, Fuijan, China

## Abstract

**Background:**

Childhood obesity is a growing global concern and is associated with cardiometabolic comorbidities in adulthood. However, the association between childhood body size and age-related macular degeneration (AMD) in late life remains to be investigated. We aimed to explore the association between childhood obesity and incident AMD and the underlying anatomical and physiobiological mechanisms.

**Methods:**

We investigated the association between childhood body size and incident AMD using multivariable Cox regression models and its relation to retinal layer thickness using linear regression. We performed four-way decomposition mediation analyses to explore the underlying mechanism. Lastly, we used univariable Mendelian randomisation (UVMR) and multivariable Mendelian randomisation (MVMR) to evaluate and differentiate the causal effect of childhood and adulthood body mass index (BMI).

**Results:**

Over a median follow-up of 12.8 years, 5026 incident AMD cases occurred among 487 009 participants. Plumper childhood body size at age 10 conferred independent risk to incident AMD in later life (adjusted hazards ratio (aHR) = 1.13; 95% confidence interval (CI) = 1.03, 1.24, *P* = 0.007) and was associated with photoreceptor outer segment layer thinning. Adulthood BMI mediated the association between childhood plumper body size and incident AMD (pure indirect effect = 33%; 95% CI = 9.9, 56.9, *P* = 0.05). Mediation analysis of adulthood physiobiological and immuno-metabolic function showed that 17 peripheral biomarkers of 7 categories significantly mediated the aforementioned pathway, with HbA1c and cystatin C showing the two largest effects. Mendelian randomisation suggested a potential causal association between childhood BMI and AMD (UVMR inverse variance weighted (IVW) odd ratio (OR) = 1.50; 95% CI = 1.09, 2.08, *P* = 0.013), independent of adulthood BMI (MVMR adulthood BMI-adjusted IVW OR = 1.29; 95% CI = 1.03, 1.61, *P* = 0.024).

**Conclusions:**

Childhood obesity may be a causal risk factor for incident AMD in later life, partially mediated by persistent obesity and physiobiological memory. Prevention of retinal degenerative diseases should therefore begin in childhood, whereby children should be encouraged and supported to maintain a normal body size.

Age-related macular degeneration (AMD) is the leading cause of irreversible vision loss in the population aged over 55 years worldwide, with the number of affected individuals projected to reach 288 million by 2040 [1,2]. The advanced stage of AMD manifests in two forms, including geographic atrophy (dry) and neovascular (wet) AMD. Currently, there is no treatment available for dry AMD, while less than half of the patients with wet AMD respond adequately to existing therapies [3]. Emphasis should therefore be put on finding risk factors and identifying high-risk populations for AMD. Apart from non-modifiable determinants, including ageing and genetic susceptibility, modifiable environmental factors, including health behaviours, obesity, and other nurture factors, have also been found to play an important role in pathogenesis [2,4].

Childhood obesity is increasing worldwide, with the global age-standardised prevalence of obesity increasing about 10-fold from 1975 to 2014 [5,6]. In the UK, the prevalence of obesity among grade 6 children (aged 10–11 years) has increased from below 4% in 1975 to 22.7% in 2020/21, with 40.9% of children being overweight or obese [7]. Childhood adiposity is associated not only with increased morbidities and premature death during childhood and adolescence [8,9], but also with irreversible changes in heart and brain structure [10,11] and an increased risk of cardiometabolic diseases [12] and neurodegenerative diseases [11] in later life. Recently, Hata and colleagues [13] demonstrated in mice that a past history of diet-induced obesity could also exacerbate retinal neuroinflammation through epigenetic reprogramming of the innate immune system, even after normalisation of metabolic abnormalities. However, despite the known association between adulthood obesity and AMD in adults [4,14], no population-level study has been conducted to investigate the long-term association between childhood obesity and incident AMD, and no Mendelian randomisation study has been performed to assess the potential causal relationship. It also remains unknown whether the potential adverse effects of childhood obesity could be reversed by achieving a healthy weight in adulthood, meaning that the role of adulthood body size in the pathway still has to be investigated.

The anatomical and physiobiological mechanisms underlying the associations of childhood adiposity and incident AMD remain elusive. Photoreceptor segment layer thinning and retinal pigment epithelium layer (RPE) cell death have been characteristic features of AMD [15,16], yet the association between adiposity and these anatomical changes is controversial [15], and the impact of childhood obesity on these changes has not been investigated. On the other hand, adiposity is related to systemic inflammation and metabolism, and several studies have demonstrated that systemic immuno-metabolic function played a role in AMD initiation and progression [17,18]. However, research on how adiposity-related systemic immuno-metabolic and physiobiological changes contribute to the adiposity-AMD association is scarce, and no population-level study has investigated the long-term effect of childhood adiposity-related systemic changes in the pathway.

Therefore, using data from 487 009 participants followed over 12 years, we investigated the association between childhood body size and AMD, adjusting for early and late life factors, and genetic predisposition to AMD. We also performed mediation analysis to discern between the effects of childhood and adulthood body size, and further used univariable and multivariable Mendelian randomisation to explore potential causal associations. Lastly, we investigated the associations between childhood body size and AMD-associated retinal layers, and explored the potential underlying systemic physiobiological and immuno-metabolic mechanisms, represented by 232 peripheral biomarkers across 24 categories, *via* a four-way decomposition mediation model.

## METHODS

### Study population

**Figure 1:**
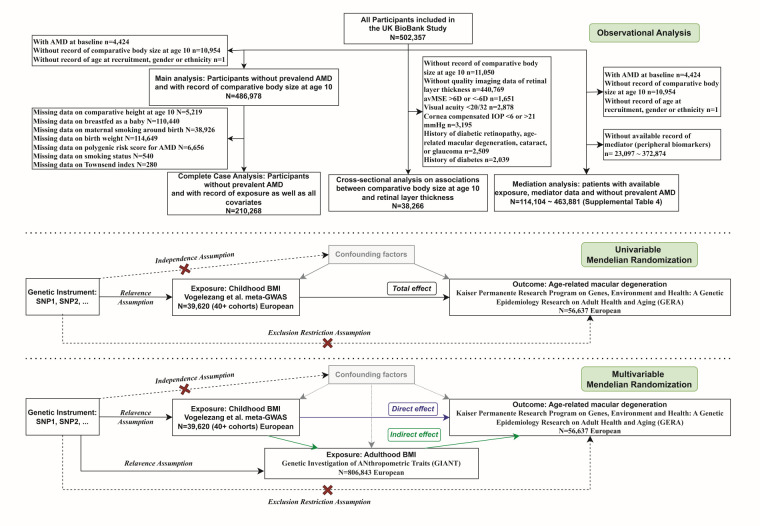
Study design and cohort creation. AMD – age-related macular degeneration, BMI – body mass index, GWAS – genome-wide association study, IVW – inverse variance weighted, MR – Mendelian randomisation, MV – multivariable, MVMR – multivariable Mendelian randomisation, SNP – single nucleotide polymorphisms, UVMR – univariable Mendelian randomisation.

The UK Biobank is a large cohort of over 500 000 participants aged 40–65 years old recruited during 2006–10 [19]. Touchscreen questionnaires and verbal interviews were used to collect sociodemographic background, lifestyle and early life factors, and health and medical history in recruitment centres. Plasma samples and imaging data were also collected at recruitment. In the main analysis of the association between childhood body size and incident AMD, we include 487 009 individuals with available records on comparative body size at age 10 and without prior AMD (**Figure 1**; Methods S1 in the **Online Supplementary Document**). The last follow-up date was defined as the earliest of the first occurrence of incident AMD, date of death, date of loss of follow-up, and last date of hospital-inpatient record follow-up, *i.e.* 1 January 2022.

### Childhood body size, covariates, and plasma biomarkers

Childhood body size was collected through touchscreen questionnaires during recruitment at the recruitment centre. Participants were presented with the question, ‘When you were 10 years old, compared to average, would you describe yourself as:’, and offered choices of ‘thinner’, ‘plumper’, ‘about average’, ‘do not know’, and ‘prefer not to answer’. During analysis, we recoded ‘do not know’ and ‘prefer not to answer’ as missing.

According to previous literature on risk factors for AMD and potential confounding early life factors [3,14], we included age at recruitment, sex, ethnicity, birth weight, maternal smoking around birth, breastfed as a baby, comparative height at age 10, polygenic risk score for AMD, Townsend index, education, prevalent diabetes, prior history of cardiovascular diseases, prior history of hyperlipidaemia, prior history of hypertension, smoking status, healthy diet, alcohol consumption, and body mass index (BMI) as covariates (Methods S1 in the **Online Supplementary Document**). Retinal layer thickness analysis further incorporated optical measures, including mean cornea-compensated intraocular pressure and mean spherical equivalent refractive error (avMSE). The collected plasma biomarkers included 31 haematology biomarkers, 4 systemic inflammation markers, 30 biochemistry biomarkers, and 168 metabolites.

### AMD diagnosis and AMD-related retinal layer thickness

We defined AMD according to self-reported health and medical history and hospital inpatient record provided by the National Health Service (NHS) England, Information and Statistics Division, Scotland, and Secure Anonymised Information Linkage, Wales. AMD identification was based on ICD-9 (code 3625), ICD-10 (code H353), and self-reported (field 20002: code 1528; field 6148: code 5) medical codes [16,20]. We defined incident AMD cases as AMD patients whose first diagnosis was after enrolment. We performed a sensitivity analysis incorporating only hospital inpatient records for AMD diagnosis to exclude potential misclassification and bias introduced by self-reported AMD history.

External limiting membrane (ELM), photoreceptor inner segment/outer segment (IS/OS) junction layer, and the RPE were segmented from optical coherence tomography (OCT) [21]. Photoreceptor inner segment layer (ELM-ISOS), photoreceptor outer segment layer (ISOS-RPE), and RPE layer were adopted as AMD-related retinal layers [22]. Details of the OCT segmentation were presented elsewhere [21]. In brief, a Topcon 3D OCT 1000 Mk2 (Topcon, Inc, Japan) was used to obtain spectral domain OCT scans, recording 512 horizontal A-scans/B-scans and 128 B-scans in a 6 × 6-mm raster pattern [23]. The Topcon Advanced Boundary Segmentation (TABS-v1.1.1) algorithm, a widely-used, accurate, and reproducible algorithm, was utilised to automatically segment all scans and provide quality score and quality control indicators [16,21,24]. The thickness of the retinal sub-layer was determined by calculating the difference between boundaries and averaged across all scans using the macula 6 grid. Eyes with an image quality score less than 45 or the poorest 20% of the inner limiting membrane indicator, the validity count indicator, or the motion indicators for the lowest correlation or the highest absolute difference were excluded for quality control of images. Then, the individual-level retinal layer thickness was determined as follows: if good-quality images were available for both eyes, the mean of the right and left eye values was used; otherwise, if a good-quality image was available for only one eye, that single eye's value was adopted. To exclude potential bias due to present systemic or ocular diseases, we excluded people from retinal layer thickness association analysis if we they had any of the following:

− a high refractive error with avMSE >6 dioptres or avMSE <−6 dioptres;

− prevalent visual impairment with logarithm of the minimum angle of resolution (logMAR) worse than 0.1;

− cornea-compensated intraocular pressure >21 mm Hg or <6 mm Hg;

− prevalent diabetic retinopathy, age-related macular degeneration, cataract, or glaucoma;

− diabetes mellitus.

### Statistical analysis

We presented categorical variables as number and percentages, and continuous variables as means and standard deviations (normal distribution) or medians and interquartile ranges (skewed distribution). We estimated the association of childhood body size with incident AMD using Cox models and with adulthood retinal layer using linear regression. We conducted multivariate imputation in chained equations for missing covariates five times in the primary analysis. We restricted our sensitivity analysis to those with complete records of covariates, and we performed subgroup analysis after multiple imputation, estimating the *P*-value for interaction using the Wald test. Lastly, as an exploratory analysis, we investigated any underlying biological mechanisms using linear and Cox regression and performed subsequent mediation analyses using Med4way [25] to estimate mediation effects (Methods S1 in the **Online Supplementary Document**). All statistical analyses were performed using Stata, version 17 (StataCorp LLC., College Station, Texas, USA).

### Mendelian randomisation

We performed two-sample Mendelian randomisation to test the causal association between childhood BMI and AMD. We detail the single-nucleotide polymorphisms (SNP) selection, summary-level genome-wide association study (GWAS) datasets, and statistical analysis in Methods S1 in the **Online Supplementary Document**. In brief, we used a recent meta-analysis conducted on a sample of 39 620 European children aged 2–10 years from 26 studies to construct instrumental variables in this study [26]. We included the SNPs that achieved genome-wide significance (*P* < 5 × 10^−8^), and we used linkage disequilibrium R^2^ (R^2^ > 0.001 within 10 000 kbs) and F-statistics (F-statistics <10) to exclude ineligible SNPs. In the univariable Mendelian randomisation (UVMR), we first utilised the GWAS catalogue to exclude SNPs that might introduce horizontal pleiotropy. These comprised SNPs significantly associated with traits influencing childhood or adult obesity, established AMD risk factors (*e.g.* smoking behaviour and dyslipidaemia), and AMD or other ocular traits (*e.g.* visual acuity and intraocular pressure) (*P*-value for confounding <5 × 10^−8^). We further excluded the SNPs with *P*-values for outcomes lower than the nominal *P*-value after Bonferroni correction (*P* < 0.05/number of SNPs). The main analysis for univariable Mendelian randomisation (UVMR) was the inverse-variance weighted (IVW) method assessing the association between childhood BMI and AMD, while the MR-Egger method, the weighted median method, and the Mendelian randomisation pleiotropy residual sum and outlier (MR-PRESSO) method acted as the sensitivity analysis. We verified the causal direction through the MR Steiger directionality and bidirectional MR. Through incorporating SNPs associated with childhood and/or adulthood BMI, we performed multivariable Mendelian Randomisation (MVMR) analysis to detect the direct and indirect causal effect of childhood BMI and distinguish between childhood and adult BMI [27]. We also performed a multivariable IVW MR, adjusting for adulthood BMI as the main analysis, and utilised the MR-Egger method to evaluate and account for horizontal pleiotropy. We set a two-sided *P*-value <0.05 as the significance threshold.

## RESULTS

### Study cohort

Among 487 009 individuals of a mean age of 56.5 years (standard deviation = 8.08) included in the study, 77 447 (15.9%) were plumper than their peers at age 10 (Table S1 in the **Online Supplementary Document**), which was equivalent to the percentage of overweight and obese children and adolescent (18.2% in girls and 15.1% in boys), defined by age-standardised BMI over one standard deviation in the UK in 1975 [6]. Those who were plumper than their peers at age 10 were younger at recruitment and had higher proportions of females, white individuals, those taller at age 10, those heavier at birth, and those more exposed to maternal smoking, and those who had been breastfed less. In their adulthood, they had lower socioeconomic status, were less educated, and had higher proportions of smokers and cardiometabolic conditions, including cardiovascular diseases, diabetes, and hyperlipidaemia.

### Childhood adiposity was associated with incident AMD and a thinner ISOS-RPE

During a median follow-up of 12.8 (interquartile range = 12.1,13.5) years, 1207 incident AMD cases occurred among individuals who were plumper at age 10, and 3819 occurred among those of average body size (**Table 1**). After multiple imputation, in the simple minimally adjusted model, plumper body size at age 10 was associated with increased risk of incident AMD in later life (adjusted hazard ratio (aHR) = 1.13; 95% confidence interval (CI) = 1.06, 1.21, *P* < 0.001). Further adjustment for early life factors, AMD polygenic risk score (PRS), and adulthood risk factors of AM did not change the association (full adjustment model: aHR = 1.13; 95% CI = 1.03, 1.24, *P* = 0.007). Incorporating self-reported AMD as incident cases or restricting the study cohort to only those with complete records of covariates likewise did not affect this association (Tables S2 and S3 in the **Online Supplementary Document**). In a subset of participants with high-quality fundus images and OCT slices and excluding participants with evidence of AMD or any drusen according to eye measures at baseline, the association remained significant and even stronger (full adjustment model: aHR = 1.39; 95% CI = 1.01, 1.93, *P* = 0.045) (Table S4 in the **Online Supplementary Document**). The absolute risk difference (ARD) of plumper childhood body size was 0.014, and the population-attributable fraction of plumper childhood body size was 2.77% (95% CI = 1.35, 4.41). Subgroup analysis showed significant multiplicative interaction between AMD PRS and childhood body size, whereby a plumper childhood body size exerted significant risk on AMD only in those with higher genetic susceptibility to AMD (AMD PRS >50% percentile). There was no significant difference in subgroups of age, sex, and ethnicity (Table S5 in the **Online Supplementary Document****)**.

**Table 1:** Adjusted Cox model for association between body size at 10 and incident AMD after multiple imputation, HR (95% CI)*

	Thinner	*P*-value	About average	Plumper	*P*-value
**Incident age-related macular degeneration**					
Number of participants	162,105		247 429	77 444	
Events/person-years	2526/2 021 722		3741/3 089 359	1182/964 877	
Model 1	1.05 (1.00, 1.11)	0.051	ref	1.13 (1.06, 1.21)	<0.001
Model 2	1.05 (0.99, 1.10)	0.082	ref	1.13 (1.06, 1.21)	<0.001
Model 3	1.04 (0.99, 1.10)	0.102	ref	1.13 (1.06, 1.21)	<0.001
Model 4	1.04 (0.98, 1.09)	0.182	ref	1.08 (1.01, 1.15)	0.022
Model 5	1.05 (0.97, 1.13)	0.200	ref	1.13 (1.03, 1.24)	0.007

ref – reference

*Values are hazard ratio (95% confidence interval) unless specified otherwise. Model 1 was adjusted for age at recruitment, gender, and ethnicity. Model 2 was further adjusted for early life factors including birth weight, maternal smoking around birth, breastfed as a baby, and comparative height at age 10 based on model 1. Model 3 was further adjusted for the genetic risk score of AMD based on model 2. Model 4 was further adjusted for other conventional risk factors of AMD based on Model 1, including Townsend index, education level, smoking status, diet, physical activity (≥500 MET min/week), alcohol (g/week), adulthood BMI category, prevalent diabetes mellitus, hypertension, cardiovascular diseases, and hyperlipidaemia. Model 5 was the full adjustment model.

We further investigated the association between childhood adiposity and AMD-related retinal layers, Among ELM-ISOS, ISOS-RPE, and RPE layer, only the ISOS-RPE was inversely associated with plumper childhood body size after full adjustment (beta = −0.17; 95% CI = −0.32, −0.03, *P* = 0.018) (**Table 2**).

**Table 2:** Adjusted Cox model for association between body size at 10 and retinal layer thickness at recruitment (aged 40–65 years) after multiple imputation*

	Thinner, *β* (95% CI)	*P*-value	About average	Plumper, *β* (95% CI)	*P*-value
**ELM-ISOS**					
Model 1	−0.00 (−0.03, 0.03)	0.935	ref	0.01 (−0.04, 0.05)	0.751
Model 2	−0.02 (−0.06, 0.02)	0.365	ref	0.01 (−0.04, 0.06)	0.742
Model 3	−0.02 (−0.06, 0.02)	0.324	ref	0.01 (−0.04, 0.06)	0.764
Model 4	−0.02 (−0.06, 0.03)	0.442	ref	0.02 (−0.04, 0.07)	0.549
**ISOS-RPE**					
Model 1	0.04 (−0.05, 0.12)	0.423	ref	−0.17 (−0.28, −0.05)	0.004
Model 2	0.06 (−0.05, 0.18)	0.304	ref	−0.19 (−0.33, −0.05)	0.008
Model 3	0.06 (−0.05, 0.18)	0.299	ref	−0.19 (−0.33, −0.05)	0.008
Model 4	0.07 (−0.09, 0.14)	0.203	ref	−0.17 (−0.32, −0.03)	0.018
**RPE**					
Model 1	−0.02 (−0.08, 0.04)	0.508	ref	−0.03 (−0.11, 0.05)	0.457
Model 2	−0.04 (−0.12, 0.04)	0.296	ref	−0.03 (−0.13, 0.07)	0.579
Model 3	−0.04 (−0.12, 0.04)	0.317	ref	−0.03 (−0.12, 0.07)	0.594
Model 4	−0.04 (−0.12, 0.04)	0.299	ref	−0.02 (−0.12, 0.07)	0.620

*β* – beta value, CI – confidence interval, ELM-ISOS – photoreceptor inner segment layer, ISOS-RPE – photoreceptor outer segment layer, ref – reference RPE – retinal pigment epithelium

*Values are hazard ratio (95% confidence interval) unless specified otherwise. Model 1 was adjusted for age at recruitment, gender, and ethnicity. Model 2 was further adjusted for early life factors, including birth weight, maternal smoking around birth, breastfed as a baby, and comparative height at age 10 on the basis of model 1. Model 3 was further adjusted for avMSE and cornea-compensated IOP. Model 4 was further adjusted for conventional risk factors of retinal degeneration, including Townsend index, education level, smoking status, alcohol consumption (g/week), healthy diet, prevalent diabetes mellitus, cardiovascular diseases, hypertension, and hyperlipidaemia.

### Adulthood immuno-metabolic and holistic biological function mediated the association

Adulthood body size, represented by continuous adulthood BMI, mediated 33.4% (95% CI = 9.9, 56.9) of the significant association between plumper childhood body size and AMD, with it still carrying significant direct excess risk. Furthermore, obesity, the conventional risk factor for AMD, mediated 28% (95% CI = 8.75, 47.2) in the pathway, and the proportion of controlled direct effect (with adulthood body size set at non-obesity level) of childhood plumper body size was 70.8% (95% CI = 25.8, 115.9) (**Table 3**).

**Table 3:** Excess relative risk proportion of direct and indirect associations of comparative plumper body size at 10 with incident age-related macular degeneration mediated by adulthood body size and peripheral biomarkers*

	Controlled direct effect, proportion (95% CI)		*P*-value		Reference interaction, proportion (95% CI)		*P*-value		Mediated interaction, proportion (95% CI)		*P*-value		Pure indirect effect, proportion (95% CI)		*P*-value	
**Adulthood body size**																	
*BMI*		63.2 (34.4, 92.1)		<0.001		0.03 (−1.8, 1.9)		0.972		3.4 (−22.3, 29.1)		0.797		33.4 (9.9, 56.9)		0.005	
*Obesity (BMI≥30)*		70.8 (25.8, 115.9)		0.002		0.66 (−25.7, 27.0)		0.961		0.56 (−21.9, 23.0)		0.961		28.0 (8.75, 47.2)		0.004	
**Liver function**																	
*Albumin*		94.3 (84.7, 104)		<0.001		−1.2 (−4.7, 2.3)		0.512		−1.5 (−8.1, 5.1)		0.664		8.3 (2.7, 13.9)		0.003	
*Gamma glutamyltransferase*		97.8 (94.7, 100.8)		<0.001		0 (−0.7, 0.6)		0.914		0.3 (−2.3, 2.9)		0.833		2.0 (0.3, 3.7)		0.022	
**Kidney function – cystatin C**		87.7 (76.1, 99.3)		<0.001		0.2 (−1.3, 1.8)		0.761		3.2 (−5.1, 11.5)		0.454		8.9 (2.6, 15.1)		0.006	
**Bone and joint function – alkaline phosphatase**		96.4 (91, 101.9)		<0.001		0.3 (−2.2, 2.9)		0.795		0.7 (−2.2, 3.6)		0.641		2.5 (0.6, 4.4)		0.009	
**Glycolysis metabolism**																	
*Glucose*		90.9 (83.4, 98.4)		<0.001		1.4 (−1.7, 4.4)		0.374		2.2 (−1.2, 5.6)		0.205		5.5 (1.8, 9.3)		0.004	
*Glycated Haemoglobin*		83 (69.7, 96.3)		<0.001		2.5 (−2, 7)		0.281		4.6 (−1.3, 10.5)		0.124		9.9 (3.5, 16.3)		0.002	
**Lipid metabolism**																	
*Cholesterol*		94.2 (87.5, 101)		<0.001		1 (−2.5, 4.6)		0.564		1.3 (−1.8, 4.5)		0.412		3.4 (0.9, 5.9)		0.008	
*LDL-direct*		96.2 (89.7, 102.7)		<0.001		0.6 (−3.9, 5.2)		0.786		0.5 (−1.6, 2.6)		0.646		2.7 (0.7, 4.7)		0.007	
**Endocrine – IGF-1**		93.5 (84.5, 102.5)		<0.001		0 (−2.3, 2.4)		0.978		0.7 (−5.7, 7)		0.837		5.8 (1.6, 10)		0.007	
**Inflammation – C-reactive protein**		87.6 (78.1, 97)		<0.001		1.4 (−1, 3.9)		0.255		4.8 (−0.9, 10.5)		0.097		6.2 (1.9, 10.5)		0.005	
**White blood cell**																	
*White blood cell (leukocyte) count*		98.2 (93.5, 102.8)		<0.001		−0.7 (−1.8, 0.4)		0.232		−2.5 (−7.8, 2.8)		0 .355		5.0 (1.3, 8.7)		0.009	
*Neutrophil count*		98.1 (93.1, 103.1)		<0.001		−0.9 (−2.8, 0.9)		0.332		−1.4 (−5.5, 2.8)		0.518		4.2 (1.1, 7.3)		0.009	
**Red blood cell**																	
*Red blood cell (erythrocyte) count*		101.1 (98.5, 103.7)		<0.001		−0.2 (−3.7, 3.4)		0.920		0 (−1.4, 1.4)		0.955		−0.9 (−1.8, 0)		0.046	
*Red blood cell (erythrocyte) distribution width*		96.6 (89.6, 103.7)		<0.001		−1.6 (−5, 1.8)		0.355		−1.5 (−6.1, 3)		0.510		6.5 (2.2, 10.8)		0.003	
*Immature reticulocyte fraction*		98.8 (93.3, 104.3)		<0.001		0.1 (−1.6, 1.7)		0.951		−5.9 (−14.3, 2.6)		0.172		7.0 (1.6, 12.4)		0.011	
*High light scatter reticulocyte percentage*		94.6 (86.3, 103)		<0.001		−0.3 (−1, 0.4)		0.373		−2.9 (−12.2, 6.5)		0.547		8.6 (2.1, 15)		0.010	
*High light scatter reticulocyte count*		95.2 (87.4, 102.9)		<0.001		−0.1 (−0.9, 0.8)		0.897		−2.7 (−12.1, 6.8)		0.578		7.5 (1.4, 13.7)		0.016	

CI – confidence interval

*All models adjusted for baseline age, gender, and ethnicity. All adulthood biomarkers were z-transformed, while skewed-distributed biomarkers were first log-transformed and then z-transformed.

We first performed linear regression to test the association between childhood body size and adulthood adiposity and peripheral biomarkers, including liver function, bone and joint function, renal function, endocrine, white and red blood cells, inflammation, lipid metabolism, glycaemic metabolism, alanine metabolism, and fluid balance. Among 232 peripheral biomarkers tested, we found significant associations in 185 biomarkers after Bonferroni correction (Table S6 in the **Online Supplementary Document**). Next, we performed Cox regression analysis to investigate the associations between these biomarkers and incident AMD (Table S7 in the **Online Supplementary Document**). We performed mediation analysis for the 29 peripheral biomarkers that showed a significant association with both childhood plumper body size and incident AMD after Bonferroni correction. Among them, 17 biomarkers from 7 categories showed significant pure indirect effects in mediating the association between plumper childhood body size and incident AMD in later life (**Table 3**; Table S8 in the **Online Supplementary Document**). We observed the maximum mediating proportion in the glycaemic metabolism category, with HbA1c mediating 9.9% (95% CI = 3.5, 16.3) of the effect. This was followed by kidney and liver function, with cystatin C mediating 8.9% (95% CI = 2.6, 15.1) and albumin mediating 8.3% (95% CI = 2.7, 13.9). Red blood cell (RBC) and inflammation biomarkers followed, with high light scatter reticulocyte percentage mediating 8.6% (95% CI = 2.1, 15) and C-reactive protein (CRP) mediating 6.2% (95% CI = 1.9, 10.5). In further adjusting the sensitivity analysis for adulthood body size and AMD PRS, nine biomarkers from seven categories still significantly mediated this association, including albumin, cystatin C, Glucose, HbA1c, cholesterol, LDL, IGF-1, CRP, and RBC distribution width (Table S9 in the **Online Supplementary Document**).

### Mendelian randomisation

**Figure 2:**
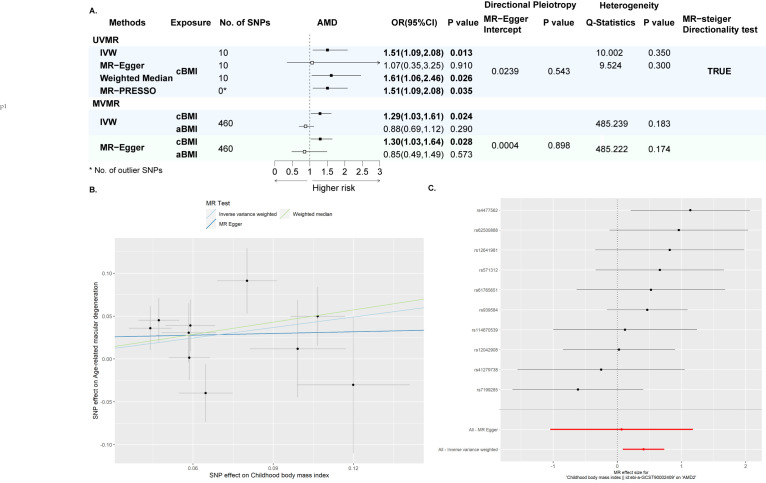
UVMR and MVMR results for the association between childhood BMI and AMD. **Panel A.** Forest plot showing results from UVMR and MVMR. AMD GWAS from the GERA consortium was used as the outcome. MR-Egger Intercept, Q-statistics, and MR-PRESSO showed there was no significant pleiotropy or heterogeneity. Circles were point estimates, solid circles showed significant results (two-sided *P* < 0.05), and hollow circles showed the opposite. **Panel B.** Scatter plot for UVMR analysis of the association between childhood BMI and AMD (GERA). **Panel C.** Forest plot for the effect of each IV on AMD. UVMR – univariate Mendelian randomisation, MVMR – multivariate Mendelian randomisation.

In the UVMR, the MR-Egger Intercept (*P* = 0.544) was insignificant and the MR-PRESSO detected no outlier SNP, showing that there was no significant pleiotropy. The Q-statistics for IVW (*P* = 0.350) and MR-Egger (*P* = 0.300) demonstrated that there was no significant heterogeneity. Therefore, we adopted IVW as the primary analysis, and significant causal association between per standard deviation increase in childhood BMI and AMD in the primary analysis (IVW odds ratio (OR) = 1.51; 95% CI = 1.09, 2.08, *P* = 0.013) (**Figure 2**; Tables S10 and S11 in the **Online Supplementary Document**). Weighted median analysis and MR-PRESSO estimates also showed significant associations, further confirming the robustness of the results. Furthermore, the reverse MR and MR Steiger directionality test confirmed the direction (Table S12 in the **Online Supplementary Document**). Sensitivity analysis using meta-analysed GWAS summary statistics for AMD showed consistent associations (UVMR-IVW: OR = 1.25; 95% CI = 1.01, 1.54, *P* = 0.038) (Table S13 in the **Online Supplementary Document**). We further performed a MVMR analysis to discern between the effect of childhood BMI and adulthood BMI(**Figure 2**) and found that the former was significantly associated with AMD (OR = 1.29; 95% CI = 1.03, 1.61, *P* = 0.024), but not the latter (OR = 0.88; 95% CI = 0.69, 1.12, *P* = 0.290). The MR-Egger intercept (*P* = 0.899) and Q-statistics (IVW *P* = 0.183, MR-Egger *P* = 0.174) showed there was no significant pleiotropy or heterogeneity.

## DISCUSSION

Based on a large prospective cohort, our findings suggest that plumper childhood body size is detrimental to incident AMD in later life. Our Mendelian randomisation analysis also demonstrated the positive causal association between childhood BMI and AMD, independent of adulthood BMI. The subgroup analysis showed that individuals with higher genetic predisposition to AMD are more vulnerable to having a plumper childhood body size, which was also related to ISOS-RPE thinning. Our mediation analysis showed that adulthood body size and adulthood physiobiological and immuno-metabolic function, represented by 17 peripheral biomarkers of 7 categories, mediated, but did not eliminate the excess risk. These findings are consistent with the hypothesis that past history of obesity has an independent and direct impact on AMD in later life, and the risk could be partially attributed to persistent body adiposity and immune-metabolic memory.

This is the first and largest prospective cohort study and Mendelian randomisation to demonstrate the positive association between plumper childhood body size and incident AMD. It is supported by previous studies on the longitudinal association between adulthood body size and incident AMD. For example, one meta-analysis showed that adulthood BMI-defined as obesity, but not overweight, was positively associated with incident AMD in Western populations, especially with late AMD [4,28]. As the childhood body size was collected through a questionnaire and was thus based on the participant’s recollection, and as it was only categorised as plumper, average, and thinner, we could not differentiate between the effect of childhood overweight and obesity. However, as the percentage of plumper childhood body size was equivalent to the age-standardised. BMI-defined overweight plus obesity prevalence rate of UK children and adolescents in 1975 [6], the actual detrimental effect of childhood obesity might be even larger. Although the ARD and population-attributable fraction of plumper childhood body size on AMD are modest, the growing global epidemic of childhood obesity, combined with its multiple adverse cardiometabolic consequences [29], underscores its significance as a major public health concern. Taken together, this evidence suggests that obesity management should be implemented across the life-course. Future studies with objectively measured childhood adiposity could help refine target childhood body size for optimal childhood and adulthood overall and retinal health.

Our retinal layer analysis showed that plumper childhood body size is associated with a thinner ISOS-RPE, but not the ELM-ISOS or the RPE layer. Photoreceptor cell loss has been observed in AMD, and photoreceptor segment layer thinning emerged decades before RPE + BM thickening and drusen deposits [16]. High-fat diet and subsequent disturbed lipid or glycaemic homeostasis could accelerate photoreceptor dysfunction, rod outer segment degeneration, and vision loss [30]. This could be partly explained by the highly metabolically demanding nature of photoreceptor functioning and outer segment renewal, and thus metabolic dysfunction may lead to retinal degeneration [31]. Further laboratory studies are warranted to investigate how the memory of adiposity and disturbed holistic metabolism influence photoreceptor cells locally and contribute to incident AMD.

Our exploration analysis and subsequent mediation analysis showed the potential underlying holistic biological mechanism of the association between childhood adiposity and incident AMD. First, we found that glycaemic metabolism, represented by HbA1c and glucose, significantly mediated the pathway. This is supported by previous studies showing the positive association between childhood obesity and diabetes [32], and between history of diabetes and incident AMD [33]. Diabetes-related functional and structural changes in choroidal circulation and RPE + Bruch’s membrane may be related to AMD [34], and disrupted blood-retina-barrier due to diabetic retinopathy could lead to increased and persistent inflammation to trigger AMD pathogenesis [35]. Second, we showed that kidney function, represented by plasma cystatin C, bridged the association. This was in line with previous studies that found how chronic kidney disease and an estimated glomerular filtration rate decrease were significantly associated with late AMD [36]. This could not only be attributed to the shared pathogenetic mechanisms such as obesity-induced systemic inflammation and up-regulated renin-angiotensin-aldosterone system, but also to heightened oxidative stress due to reduced glomerular filtration of free radical-generating wastes, accelerating degeneration and calcification of Bruch’s membrane and choroidal neovascularisation [37]. Third, we observed that liver function also played an important role, which aligns with a previous cross-sectional study which found a relationship between serum gamma-glutamyl transferase level and AMD. This can be partially explained by the prominent role liver-produced complement factor H-related protein 4 plays in AMD pathogenesis [38]. Fourth, systemic inflammation, including C-reactive protein and white blood cells, also mediated the pathway, which is substantiated by populational and laboratory literature demonstrating inflammation as a critical pathogenic component of wet and dry AMD [39]. However, studies on how obesity history contributes to later retinal biological and immune-metabolic functions to impact retinal degeneration are still warranted.

The strengths of our study include its large sample size, its prospective design, and our investigation of underlying anatomical and biological mechanisms. However, this research also has some limitations. First, the cohort was largely composed of Caucasians aged 40–70 years, so generalisation into other ethnicities and age groups is not possible. Second, as childhood body size was collected through patients’ recall in later life, misclassification of body size may be possible due to recall bias. However, childhood body sizes obtained through this way were found to be valid and comparable to those collected through objective measures [40–42], and the Mendelian randomisation based on objectively measured childhood BMI in our study showed the same results. Third, the observational nature of this study prevented us from accounting for all residual confounding regarding childhood body size and AMD. Fourth, the identification of AMD in the UK Biobank study was based on ICD codes from hospital inpatient records from NHS, so asymptomatic AMD cases may be underdiagnosed due to a lack of imaging data.

## CONCLUSIONS

Childhood adiposity plays an independent detrimental role in incident AMD in later life, partially mediated by adulthood adiposity and holistic metabolism and inflammation. Therefore, interventions targeting healthy body size should begin early since childhood to help prevent adverse health outcomes in adulthood and old age.

## Additional material


Online Supplementary Document
