# Supplementary material for: Childhood obesity directly increases age-related macular degeneration risk: the role of physiobiological and immune-metabolic function
Source: J Glob Health. 2025 Jun 27;15:04164. doi: 10.7189/jogh.15.04164 (PMC12201934; doi:10.7189/jogh.15.04164)
Supplement: Online Supplementary Document [file jogh-15-04164-s001.zip › jogh-15-04164-s001.pdf]

Cite as: Zhang X, Huang Y, Ma M, Zhou C, Jiang Y, Zhang Z, Zhu X, Li C, Xu X, Fan Y, Han C, Zheng Z, Zhao S. Childhood obesity directly increases age-related macular degeneration risk: the role of physiobiological and immune-metabolic function. J Glob Health. 2025;15:04164.

Supplemental Methods:

Observational Study

Study population

In the main analysis of the association between childhood body size and incident AMD, those with available record on comparative body size at age 10 and without prior AMD were included in the primary analysis, which was 487,009 participants. [Figure 1] Exploration and mediation analysis for potential mediating peripheral biomarkers were performed in those with available plasma biomarker or metabolomics data, available childhood body size, and without prevalent AMD. [Supplemental Table 4]

Covariates:

Age at recruitment, sex (male or female), ethnicity (white or other), birth weight (g, per SD increase), maternal smoking around birth (yes or no), breastfed as a baby (yes or no), comparative height at age 10 (shorter, average, or taller), polygenic risk score for AMD, Townsend index, education (college/university or below), prevalent diabetes (yes or no), prior history of cardiovascular diseases (yes or no), prior history of hyperlipidemia (yes or no), prior history of hypertension (yes or no), smoking status (current, previous or never), healthy diet (yes or no), alcohol consumption (per SD increase in units per week), and body mass index (underweight, normal, overweight, or obese) were included as covariates according to prior literatures on risk factors for AMD and potential confounding early life factors. Retinal layer thickness association analysis further incorporated optical measures including mean cornea compensated intraocular pressure (IOPcc), and mean spherical equivalent refractive error (avMSE).

Polygenic risk score for AMD (Field 26204) was constructed using genotype and imputation data generated from plasma samples collected at recruitment. PRS construction was supported by external GWAS meta-analysis incorporating the Electronic Medical Records and Genomics (eMERGE)-I, eMERGE-II and eMERGE-III, and details were described elsewhere. [23] Townsend index was derived according to postcode based on preceding national census output areas, and a positive value denoted more materially deprived. Prevalent diabetes was defined by self-reported history of diabetes, hospital inpatient diagnosis of all kinds of diabetes except for gestational diabetes before recruitment, use of antidiabetic medications or insulin, or baseline HbA1c>=48mmol/mol. (microvascular burden and incident heart failure among middle-aged and older adults with Type 1 or Type 2 diabetes) Prior history of cardiovascular disease included self-reported or hospital inpatient disease record of myocardial infarction, angina pectoris, unstable angina, or cardiac arrest, or hospital inpatient operation record of percutaneous coronary intervention or coronary artery replacement. History of hypertension was identified according to self-reported prior doctor-diagnosed hypertension, antihypertensive medication, or a blood pressure >=140/90 mmHg. History of hyperlipidemia was identified according to self-reported prior doctor-diagnosed hyperlipidemia, lipid-lowering medication, or plasma dyslipidemia at recruitment: triglycerides > 2.30mmol/L, total cholesterol > 6.21 mmol/L, low-density lipoprotein cholesterol > 4.10mmol/L, high-density lipoprotein cholesterol < 1.00mmol/L (male) or <1.30mmol/L (female). [1] Body mass index (BMI) was used to define adulthood body size. BMI (kg/m^2) = Weight (kg) / height ^2 (m^2) was used to calculate BMI for those with complete record of weight and height. BMI was z-transformed to estimate the effect of per-SD increase in BMI. BMI was also recategorized according to WHO recommendation: underweight (BMI<18), normal (BMI >=18-25), overweight (BMI >=25-30), obese (BMI>=30).

Plasma biomarkers: blood count, inflammatory markers, blood biochemistry, and metabolomics

Baseline blood samples of UKB participants were collected at the initial assessment center visit. Details of sample collection and storage were described elsewhere. [2]Thirty-one hematology biomarkers were analyzed at the UK Biobank central laboratory within 24 hours of collection using a Beckman Coulter LH750 Hematology Analyzer.[3] Four systemic inflammation markers were derived from blood cells counts: neutrophil-to-lymphocyte ratio (MLR, neutrophil/lymphocytes), lymphocyte-to-monocyte ratio (LMR, lymphocytes/monocyte), platelet-to-lymphocyte ratio (PLR, platelets/lymphocytes), and systemic immune-inflammation index (SII, neutrophils*platelets/lymphocytes). [4,5] Thirty blood biochemistry biomarkers were analyzed using DiaSorin Liaison XL, Beckman Coulter DXI800, Beckman Coulter AU5800, and Siemens Advia 1800.[6] 168 metabolites, covering amino acids, lipids, lipoproteins, cholesterol subtypes, and inflammation markers, were directly measured using a high-throughput nuclear magnetic resonance (NMR)-based metabolites profiling platform developed by Nightingale Health Ltd. [7,8] All peripheral biomarkers were z-transformed, and skewed distributed biomarkers were log-transformed then z-transformed. Details are provided in Supplemental Methods.

Mediation Analysis

First, linear regression was used to assess association between plumper body size and peripheral biomarkers. Each regression was adjusted for age, sex and ethnic. Then, cox regression was performed for those who passed Bonferroni-adjusted p value. Last, we performed mediation analyses for those variables significantly and consistently related to childhood body size and incident AMD using Med4way.[13] The total effect of childhood body size on incident AMD was decomposed into 4 potential causal pathways: (1) controlled direct effect, (2) pure indirect effect, (3) reference interaction effect, and (4) mediated interaction effect. The proportion was defined as the ratio of each effect to the total effect at mean levels of interested mediators. Models were adjusted for age, sex, and ethnic background. Sensitivity analysis was performed further adjusting for adult obesity (BMI>=30) and polygenic risk score for AMD.

Mendelian randomization

Mendelian randomization uses genetic variants as IVs to estimate causal effect of exposure on outcome, unbiased by residual confounding and reverse causation. Three major assumptions must be satisfied to perform MR analysis, including relevance assumption, independence assumption, and assumption of exclusion restriction. [14]

A recent meta-analysis using 39,620 European children aged 2-10 years from 26 studies was used to construct instrumental variables (IVs) in this study. [15] [eTable 7-8] Age- and sex-adjusted BMI Standard Deviation Scores (SDS) were used in the meta-GWAS. Full details of IV associations with exposure and outcome were presented in eTable 5. Single nucleotide polymorphisms (SNPs) that achieved genome-wide significance ($P<5*10^{-8}$) were included, and SNPs at the same locus (linkage disequilibrium R2>0.001 within 10,000kbs) were excluded. R2 and F-statistics were calculated and SNPs with F-statistics<10 were excluded to avoid weak instrument bias. We further used GWAS Catalog to exclude SNPs that have significant associations ($P_{confounder} < 5*10^{-8}$) with AMD and conventional risk factors of AMD, including smoking behavior and hyperlipidemia. (https://www.ebi.ac.uk/gwas/, accessed on 13 May 2024) We also excluded SNPs with $P_{outcome}$ values lower than the nominal P-value after Bonferroni correction (P<0.05/N, SNPs) as they were considered directly related to AMD. We also harmonized the effects of IVs on exposure and outcome and excluded palindromic SNPs and SNPs with incompatible alleles. In MVMR, adulthood BMI dataset source was from the Genetic Investigation of Anthropometric Traits consortium (GIANT) (N=806,834).[16]

The AMD GWAS data was derived in the Kaiser Permanente Research Program on Genes, Environment and Health: A Genetic Epidemiology Research on Adult

Health and Aging (GERA) study (cases/controls=3,685/52,952).[17] In GERA study, AMD was defined according to ICD-9-CM codes: 362.5, 362.50, 362.51, 362.52, 362.57. In FinnGen consortium, The FinnGen data used ICD 8-10 codes to identify both dry and wet AMD, and details was described elsewhere.[18] Sensitivity analysis was performed using meta-analysis GWAS of AMD. [eTable 10] As the GERA study was conducted in United State of America and 6031 participants of the exposure meta-GWAS was American, the sample overlap is no more than 10% in the primary analysis even if all American participants were overlapped. To further account for potential sample overlap, sensitivity analysis was performed using meta-GWAS of AMD. We used METAL to perform meta-analysis based on summary-level data from two cohorts: the primary GERA study, and the R9 release of FinnGen consortium (cases/controls=8,913/348,936).[19] The sensitivity analysis using AMD meta-GWAS should have far less sample overlap. [20]

Inverse-variance weighted (IVW) method was adopted as the main analysis, a method providing precise and efficient estimate when there is no significant heterogeneity or pleiotropy.[21] Therefore, we further performed three sensitivity analysis to test and account for heterogeneity and pleiotropy. The MR-Egger method implicates directional pleiotropy thorough non-zero intercept and provides pleiotropy-corrected causal estimates, though it's underpowered when there was no pleiotropy existence. [22,23] The Weighted Median method was robust even if up to 50% of the IVs were invalid. [24] The Mendelian Randomization Pleiotropy RESidual Sum and Outlier (MR-PRESSO) method generates estimated robust to horizontal pleiotropy through exclusion of outlier SNPs. [25]

All analysis were performed using R version 4.2.1, and “TwoSampleMR” (ver. 0.5.6)), “MRPRESSO” (ver. 1.0), “Mendelian Randomization” (ver. 0.6.0), and “MVMR” (ver. 0.3) R packages were used.

Reference:

1. Kopin L, Lowenstein CJ. Dyslipidemia. *Ann Intern Med*. 2017;167(11):ITC81-ITC96. doi:10.7326/AITC201712050
2. Elliott P, Peakman TC, on behalf of UK Biobank. The UK Biobank sample handling and storage protocol for the collection, processing and archiving of human blood and urine. *International Journal of Epidemiology*. 2008;37(2):234-244. doi:10.1093/ije/dym276
3. Hematology : Resource 1453. Accessed January 18, 2024. https://biobank.ndph.ox.ac.uk/showcase/refer.cgi?id=1453
4. Yang X, Zhao S, Wang S, et al. Systemic inflammation indicators and risk of incident arrhythmias in 478,524 individuals: evidence from the UK Biobank cohort. *BMC Med*. 2023;21:76. doi:10.1186/s12916-023-02770-5
5. Nøst TH, Alcala K, Urbarova I, et al. Systemic inflammation markers and cancer incidence in the UK Biobank. *Eur J Epidemiol*. 2021;36(8):841-848. doi:10.1007/s10654-021-00752-6
6. Biochemistry: Resource 1227. Accessed May 13, 2024. https://biobank.ndph.ox.ac.uk/showcase/refer.cgi?id=1227
7. Metabolomics: Resource 3000. Accessed May 13, 2024. https://biobank.ndph.ox.ac.uk/showcase/refer.cgi?id=3000
8. Würtz P, Havulinna AS, Soininen P, et al. Metabolite Profiling and Cardiovascular Event Risk. *Circulation*. 2015;131(9):774-785. doi:10.1161/CIRCULATIONAHA.114.013116
9. Patel PJ, Foster PJ, Grossi CM, et al. Spectral-Domain Optical Coherence Tomography Imaging in 67 321 Adults: Associations with Macular Thickness in the UK Biobank Study. *Ophthalmology*. 2016;123(4):829-840. doi:10.1016/j.ophtha.2015.11.009
10. Zekavat SM, Sekimitsu S, Ye Y, et al. Photoreceptor layer thinning is an early biomarker for age-related macular degeneration: Epidemiological and genetic evidence from UK Biobank optical coherence tomography data. *Ophthalmology*. 2022;129(6):694-707. doi:10.1016/j.ophtha.2022.02.001
11. Khawaja AP, Chua S, Hysi PG, et al. Comparison of Associations with Different Macular Inner Retinal Thickness Parameters in a Large Cohort: The UK Biobank. *Ophthalmology*. 2020;127(1):62-71. doi:10.1016/j.ophtha.2019.08.015
12. Ko F, Foster PJ, Strouthidis NG, et al. Associations with Retinal Pigment Epithelium Thickness Measures in a Large Cohort: Results from the UK Biobank. *Ophthalmology*. 2017;124(1):105-117. doi:10.1016/j.ophtha.2016.07.033
13. Discacciati A, Bellavia A, Lee JJ, Mazumdar M, Valeri L. Med4way: a Stata command to investigate mediating and interactive mechanisms using the four-way effect decomposition. *International Journal of Epidemiology*. 2019;48(1):15-20. doi:10.1093/ije/dyy236
14. de Leeuw C, Savage J, Bucur IG, Heskes T, Posthuma D. Understanding the assumptions underlying Mendelian randomization. *Eur J Hum Genet*. 2022;30(6):653-660. doi:10.1038/s41431-022-01038-5
15. Vogelezang S, Bradfield JP, Ahluwalia TS, et al. Novel loci for childhood body mass index and shared heritability with adult cardiometabolic traits. *PLoS Genet*. 2020;16(10):e1008718. doi:10.1371/journal.pgen.1008718
16. Pulit SL, Stoneman C, Morris AP, et al. Meta-analysis of genome-wide association studies for body fat distribution in 694 649 individuals of European ancestry. *Hum Mol Genet*. 2019;28(1):166-174. doi:10.1093/hmg/ddy327
17. Guindo-Martínez M, Amela R, Bonàs-Guarch S, et al. The impact of non-additive genetic associations on age-related complex diseases. *Nat Commun*. 2021;12:2436. doi:10.1038/s41467-021-21952-4
18. Kurki MI, Karjalainen J, Palta P, et al. FinnGen provides genetic insights from a well-phenotyped isolated population. *Nature*. 2023;613(7944):508-518. doi:10.1038/s41586-022-05473-8
19. Willer CJ, Li Y, Abecasis GR. METAL: fast and efficient meta-analysis of genomewide association scans. *Bioinformatics*. 2010;26(17):2190-2191. doi:10.1093/bioinformatics/btq340
20. Burgess S, Davies NM, Thompson SG. Bias due to participant overlap in two-sample Mendelian randomization. *Genet Epidemiol*. 2016;40(7):597-608. doi:10.1002/gepi.21998
21. Burgess S, Davey Smith G, Davies NM, et al. Guidelines for performing Mendelian randomization investigations: update for summer 2023. *Wellcome Open Res*. 2023;4:186. doi:10.12688/wellcomeopenres.15555.3
22. Bowden J, Davey Smith G, Burgess S. Mendelian randomization with invalid instruments: effect estimation and bias detection through Egger regression. *Int J Epidemiol*. 2015;44(2):512-525. doi:10.1093/ije/dyv080
23. Burgess S, Thompson SG. Interpreting findings from Mendelian randomization using the MR-Egger method. *Eur J Epidemiol*. 2017;32(5):377-389. doi:10.1007/s10654-017-0255-x
24. Bowden J, Davey Smith G, Haycock PC, Burgess S. Consistent Estimation in Mendelian Randomization with Some Invalid Instruments Using a Weighted Median Estimator. *Genet Epidemiol*. 2016;40(4):304-314. doi:10.1002/gepi.21965
25. Verbanck M, Chen CY, Neale B, Do R. Detection of widespread horizontal pleiotropy in causal relationships inferred from Mendelian randomization between complex traits and diseases. *Nat Genet*. 2018;50(5):693-698. doi:10.1038/s41588-018-0099-7

Table S1. Baseline characteristics stratified by body size at age 10.

Comparative body size at 10	Overall *N= 486,978*	About average *N=247,429*	Thinner *N=162,105*	Plumper *N=77,444*	P-value
Age, year (mean (SD))	56.48 (8.08)	56.70 (8.08)	56.47 (8.14)	55.76 (7.94)	<0.001
Sex (%)					<0.001
Female	265,624 (54.5)	134091 (54.2)	84439 (52.1)	47094 (60.8)	
Male	221,354 (45.5)	113338 (45.8)	77666 (47.9)	30350 (39.2)	
Ethnicity (%)					<0.001
White	460,493 (94.6)	235367 (95.1)	151488 (93.5)	73638 (95.1)	
Others	26,485 (5.4)	12062 (4.9)	10617 (6.5)	3806 (4.9)	
Maternal smoking around birth (%)					<0.001
No	297652 (70.6)	153960 (72.3)	99082 (70.1)	44610 (66.4)	
Yes	123843 (29.4)	59075 (27.7)	42203 (29.9)	22565 (33.6)	
Missing	13.4	12.8	13.9	13.3	
Breastfed as a baby (%)					<0.001
No	103881 (27.7)	50302 (26.4)	35116 (28.3)	18463 (30.6)	
Yes	270699 (72.3)	140051 (73.6)	88812 (71.7)	41836 (69.4)	
Missing	23.1	23.6	23.1	22.1	
Comparative height at 10 (%)					<0.001
About average	261924 (54.4)	153143 (62.5)	70787 (44.2)	37994 (49.8)	
Shorter than average	98160 (20.4)	39191 (16.0)	45159 (28.2)	13810 (18.1)	
Taller than average	121675 (25.3)	52890 (21.6)	44308 (27.6)	24477 (32.1)	
Missing	1.1	1.1	0.9	1.5	
Birthweight, g (mean (SD))	3319.85 (666.83)	3356.49 (641.69)	3220.24 (677.90)	3402.54 (697.05)	<0.001
Missing (%)	44.3	45.6	44.2	41.8	
Polygenic risk score for AMD (mean (SD))	0.13 (1.03)	0.13 (1.03)	0.13 (1.03)	0.12 (1.03)	0.056
Missing (%)	3.1	3.1	3.2	3.4	
Townsend index (mean (SD))	-1.32 (3.07)	-1.42 (3.02)	-1.25 (3.12)	-1.17 (3.14)	<0.001
Missing (%)	0.1	0.1	0.1	0.1	
Education (%)					<0.001
University/college or equivalent	157569 (32.4)	80452 (32.5)	51536 (31.8)	25581 (33.0)	
Below university/college	329409 (67.6)	166977 (67.5)	110569 (68.2)	51863 (67.0)	
Smoker status (%)					<0.001
Never	265513 (54.7)	136182 (55.2)	88308 (54.7)	41023 (53.2)	
Former/current	219743 (45.3)	110408 (44.8)	73197 (45.3)	36138 (46.8)	
Missing	0.4	0.4	0.3	0.4	
Diet (%)					<0.001
Unhealthy	441180 (90.6)	224584 (90.8)	146719 (90.5)	69877 (90.2)	
Healthy	45798 (9.4)	22845 (9.2)	15386 (9.5)	7567 (9.8)	
Alcohol, g/week	92.84 (77.79)	93.93 (77.50)	93.46 (78.14)	88.09 (77.82)	<0.001
Physical activity >=500 METmin/week					<0.001
No	61701 (12.7)	29315 (11.8)	21404 (13.2)	10982 (14.2)	
Yes	331373 (68.0)	170313 (68.8)	109715 (67.7)	51345 (66.3)	
Missing (%)	19.3	19.4	19.1	19.5	
BMI status					<0.001
Underweight (<18kg/m2)	2500 (0.5)	1046 (0.4)	1277 (0.8)	177 (0.2)	
Normal (18-25kg/m2)	158024 (32.6)	79918 (32.5)	63507 (39.4)	14599 (19.0)	
Overweight (25-30kg/m2)	205721 (42.5)	109320 (44.4)	66327 (41.1)	30074 (39.1)	
Obesity (>30kg/m2)	118211 (24.4)	55910 (22.7)	30145 (18.7)	32156 (41.8)	
Missing (%)	0.5	0.5	0.5	0.6	
History of cardiovascular disease (%)					<0.001
No	454490 (93.3)	231853 (93.7)	150567 (92.9)	72070 (93.1)	
Yes	32488 (6.7)	15576 (6.3)	11538 (7.1)	5374 (6.9)	
History of diabetes mellitus (%)					<0.001
No	457202 (93.9)	234385 (94.7)	151532 (93.5)	71285 (92.0)	
Yes	29776 (6.1)	13044 (5.3)	10573 (6.5)	6159 (8.0)	
History of hyperlipidemia (%)					<0.001
No	251412 (51.6)	131702 (53.2)	80479 (49.6)	39231 (50.7)	
Yes	235566 (48.4)	115727 (46.8)	81626 (50.4)	38213 (49.3)	
History of hypertension (%)					<0.001
No	220938 (45.4)	113786 (46.0)	73111 (45.1)	34041 (44.0)	
Yes	266040 (54.6)	133643 (54.0)	88994 (54.9)	43403 (56.0)	

SD, standard deviation.

Table S2. Adjusted Cox model for association between body size at 10 and incident AMD (hospital inpatient + self-reported AMD) after multiple imputation.

Comparative body size at age 10	Thinner HR (95%CI)	P-value	About Average HR (95%CI)	Plumper HR (95%CI)	P-value
Incident AMD					
No. of participants	162,105		247,429	77,444	
Events/Person-years	2,586/2,021,282		3,819/3,088,754	1,207/964,688	
Model 1	1.05(1.00, 1.11)	0.037	1[Ref]	1.13(1.06, 1.21)	<0.001
Model 2	1.05(1.00, 1.10)	0.062	1[Ref]	1.13(1.06, 1.20)	<0.001
Model 3	1.05(0.99, 1.10)	0.078	1[Ref]	1.13(1.06, 1.21)	<0.001
Model 4	1.04(0.99, 1.09)	0.131	1[Ref]	1.08(1.01, 1.15)	0.021
Model 5	1.06(0.99, 1.15)	0.097	1[Ref]	1.13(1.03, 1.24)	0.009

Model 1 adjusted for age at recruitment, gender and ethnicity.

Model 2 further adjusted for early life factors including birth weight, maternal smoking around birth, breastfed as a baby, and comparative height at age 10 on the basis of model 1.

Model 3 further adjusted for genetic risk score of AMD on the basis of model 2.

Model 4 further adjusted for other conventional risk factors of AMD on the basis of Model 1, including Townsend index, education level, smoking status, diet, physical activity (>=500METmin/week), alcohol (g/week), adulthood BMI category, prevalent diabetes mellitus, hypertension, cardiovascular diseases and hyperlipidemia.

Model 5 was the full adjustment model.

Table S3. Adjusted Cox model for association between body size at 10 and incident AMD in patients with complete data on covariates.

**Comparative body size at age 10**	**Thinner HR (95%CI)**	**P-value**	**About Average HR (95%CI)**	**Plumper HR (95%CI)**	**P-value**
Incident AMD					
No. of participants	70,725		108,802	35,520	
Events/Person-years	1,088/877,954		1,621/1,360,698	572/442,680	
Model 1	1.06(0.99, 1.15)	0.113	1[Ref]	1.19(1.08, 1.31)	<0.001
Model 2	1.05(0.96, 1.14)	0.291	1[Ref]	1.17(1.06, 1.30)	0.002
Model 3	1.04(0.95, 1.13)	0.381	1[Ref]	1.17(1.05, 1.30)	0.003
Model 4	1.05(0.97, 1.14)	0.196	1[Ref]	1.13(1.03, 1.25)	0.012
Model 5	1.03(0.94, 1.12)	0.546	1[Ref]	1.12(1.01, 1.24)	0.037

Model 1 adjusted for age at recruitment, gender and ethnicity.

Model 2 further adjusted for early life factors including birth weight, maternal smoking around birth, breastfed as a baby, and comparative height at age 10 on the basis of model 1.

Model 3 further adjusted for genetic risk score of AMD on the basis of model 2.

Model 4 further adjusted for other conventional risk factors of AMD on the basis of Model 1, including Townsend index, education level, smoking status, diet, physical activity (>=500METmin/week), alcohol (g/week), adulthood BMI category, prevalent diabetes mellitus, hypertension, cardiovascular diseases and hyperlipidemia.

Model 5 was the full adjustment model.

Table S4. Adjusted Cox model for association between body size at 10 and incident AMD in patients with OCT and Fundus image and without AMD at baseline.

Comparative body size at age 10	Thinner HR (95%CI)	P-value	About Average HR (95%CI)	Plumper HR (95%CI)	P-value
Incident AMD					
No. of participants	16,441		25,195	7,944	
Events/Person-years	95/187,551		127/287,706	55/90,538	
Model 1	1.19(0.91,1.56)	0.192	1[Ref]	1.48(1.08,2.03)	**0.015**
Model 2	1.18(0.90,1.55)	0.230	1[Ref]	1.48(1.07,2.03)	**0.017**
Model 3	1.18(0.90,1.55)	0.232	1[Ref]	1.28(1.07,2.04)	**0.016**
Model 4	1.18(0.90,1.54)	0.230	1[Ref]	1.39(1.01,1.92)	**0.044**
Model 5	1.17(0.89,1.53)	0.263	1[Ref]	1.39(1.01,1.93)	**0.045**

Model 1 adjusted for age at recruitment, gender and ethnicity.

Model 2 further adjusted for early life factors including birth weight, maternal smoking around birth, breastfed as a baby, and comparative height at age 10 on the basis of model 1.

Model 3 further adjusted for genetic risk score of AMD on the basis of model 2.

Model 4 further adjusted for other conventional risk factors of AMD on the basis of Model 1, including Townsend index, education level, smoking status, diet, physical activity (>=500METmin/week), alcohol (g/week), adulthood BMI category, prevalent diabetes mellitus, hypertension, cardiovascular diseases and hyperlipidemia.

Model 5 was the full adjustment model.

Table S5. Subgroup stratified adjusted Cox model for association between body size at 10 and incident AMD after multiple imputation.

Comparative body size at age 10	Number	Percentage	HR (95%CI)	P-value	P for interaction
Age					0.751
<60	277,793	57.0%			
Thinner vs Average			1.06 (0.95, 1.19)	0.268	
Plumper vs Average			1.04 (0.91, 1.19)	0.565	
>=60	209,185	43.0%			
Thinner vs Average			1.02 (0.96, 1.08)	0.510	
Plumper vs Average			1.09 (1.01, 1.18)	**0.022**	
Gender					0.091
Female	265,624	54.5%			
Thinner vs Average			1.05 (0.98, 1.12)	0.166	
Plumper vs Average			1.13 (1.04, 1.23)	**0.003**	
Male	221354	45.5%			
Thinner vs Average			0.99 (0.92, 1.08)	0.902	
Plumper vs Average			0.99 (0.88, 1.11)	0.857	
Ethnicity					0.925
White	460493	94.6%			
Thinner vs Average			1.03 (0.98, 1.09)	0.262	
Plumper vs Average			1.08 (1.01, 1.16)	**0.023**	
Others	26,485	5.4%			
Thinner vs Average			0.98 (0.78, 1.22)	0.849	
Plumper vs Average			1.06 (0.78, 1.44)	0.699	
Adulthood body size					0.869
BMI < 30	366,245	75.2%			
Thinner vs Average			1.03 (0.97, 1.10)	0.276	
Plumper vs Average			1.09 (1.00, 1.18)	0.053	
BMI >=30	120,733	24.8%			
Thinner vs Average			1.01 (0.91, 1.11)	0.917	
Plumper vs Average			1.06 (0.96, 1.18)	0.247	
AMD PRS					**<0.001**
<50 percentile	235,847	50.0%			
Thinner vs Average			1.02 (0.94, 1.11)	0.654	
Plumper vs Average			1.08 (0.97, 1.11)	0.158	
>=50 percentile	235,846	50.0%			
Thinner vs Average			1.03 (0.96, 1.10)	0.447	
Plumper vs Average			1.10 (1.01, 1.20)	**0.023**	

Models adjusted for age at recruitment, gender, ethnicity, early life factors including birth weight, maternal smoking around birth, breastfed as a baby, and comparative height at age 10, genetic risk score of AMD, Townsend index, education level, smoking status, diet, physical activity (>=500METmin/week), alcohol (g/week), prevalent diabetes mellitus, hypertension, cardiovascular diseases and hyperlipidemia.

STROBE Statement—checklist of items that should be included in reports of observational studies

	Item No	Recommendation	Page No
**Title and abstract**	1	(*a*) Indicate the study's design with a commonly used term in the title or the abstract	2
		(*b*) Provide in the abstract an informative and balanced summary of what was done and what was found	2
**Introduction**			
Background/rationale	2	Explain the scientific background and rationale for the investigation being reported	3-4
Objectives	3	State specific objectives, including any prespecified hypotheses	3-4
**Methods**			
Study design	4	Present key elements of study design early in the paper	4-5
Setting	5	Describe the setting, locations, and relevant dates, including periods of recruitment, exposure, follow-up, and data collection	4, Supplemental Methods
Participants	6	(*a*) *Cohort study*—Give the eligibility criteria, and the sources and methods of selection of participants. Describe methods of follow-up<br>*Case-control study*—Give the eligibility criteria, and the sources and methods of case ascertainment and control selection. Give the rationale for the choice of cases and controls<br>*Cross-sectional study*—Give the eligibility criteria, and the sources and methods of selection of participants	4, Supplemental Methods
		(*b*) *Cohort study*—For matched studies, give matching criteria and number of exposed and unexposed<br>*Case-control study*—For matched studies, give matching criteria and the number of controls per case	NA
Variables	7	Clearly define all outcomes, exposures, predictors, potential confounders, and effect modifiers. Give diagnostic criteria, if applicable	4-5
Data sources/ measurement	8*	For each variable of interest, give sources of data and details of methods of assessment (measurement). Describe comparability of assessment methods if there is more than one group	*4*
Bias	9	Describe any efforts to address potential sources of bias	5-6, Supplemental Methods
Study size	10	Explain how the study size was arrived at	5
Quantitative variables	11	Explain how quantitative variables were handled in the analyses. If applicable, describe which groupings were chosen and why	5, Supplemental Methods
Statistical methods	12	(*a*) Describe all statistical methods, including those used to control for confounding	5, Supplemental Methods
		(*b*) Describe any methods used to examine subgroups and interactions	NA
		(*c*) Explain how missing data were addressed	5
		(*d*) *Cohort study*—If applicable, explain how loss to follow-up was addressed<br>*Case-control study*—If applicable, explain how matching of cases and controls was addressed<br>*Cross-sectional study*—If applicable, describe analytical methods taking account of sampling strategy	4
		(*e*) Describe any sensitivity analyses	7

Continued on next page

Results

Participants	13*	(a) Report numbers of individuals at each stage of study—eg numbers potentially eligible, examined for eligibility, confirmed eligible, included in the study, completing follow-up, and analysed	3, Fig.1
		(b) Give reasons for non-participation at each stage	3, Fig.1
		(c) Consider use of a flow diagram	Fig.1
Descriptive data	14*	(a) Give characteristics of study participants (eg demographic, clinical, social) and information on exposures and potential confounders	6
		(b) Indicate number of participants with missing data for each variable of interest	Sup.T1
		(c) *Cohort study*—Summarise follow-up time (eg, average and total amount)	6
Outcome data	15*	*Cohort study*—Report numbers of outcome events or summary measures over time	*6*
		Case-control study—Report numbers in each exposure category, or summary measures of exposure	
		Cross-sectional study—Report numbers of outcome events or summary measures	
Main results	16	(*a*) Give unadjusted estimates and, if applicable, confounder-adjusted estimates and their precision (eg, 95% confidence interval). Make clear which confounders were adjusted for and why they were included	6-7
		(*b*) Report category boundaries when continuous variables were categorized	NA
		(*c*) If relevant, consider translating estimates of relative risk into absolute risk for a meaningful time period	6
Other analyses	17	Report other analyses done—eg analyses of subgroups and interactions, and sensitivity analyses	6-7

Discussion

Key results	18	Summarise key results with reference to study objectives	8
Limitations	19	Discuss limitations of the study, taking into account sources of potential bias or imprecision. Discuss both direction and magnitude of any potential bias	9
Interpretation	20	Give a cautious overall interpretation of results considering objectives, limitations, multiplicity of analyses, results from similar studies, and other relevant evidence	9-10
Generalisability	21	Discuss the generalisability (external validity) of the study results	9

Other information

Funding	22	Give the source of funding and the role of the funders for the present study and, if applicable, for the original study on which the present article is based	10

*Give information separately for cases and controls in case-control studies and, if applicable, for exposed and unexposed groups in cohort and cross-sectional studies.

Note: An Explanation and Elaboration article discusses each checklist item and gives methodological background and published examples of transparent reporting. The STROBE checklist is best used in conjunction with this article (freely available on the Web sites of PLoS Medicine at http://www.plosmedicine.org/, Annals of Internal Medicine at http://www.annals.org/, and Epidemiology at http://www.epidem.com/). Information on the STROBE Initiative is available at www.strobe-statement.org.

STROBE-MR checklist of recommended items to address in reports of Mendelian randomization studies[1 2]

Item No.	Section		Checklist item	Page No.	Relevant text from manuscript
1	**TITLE and ABSTRACT**		Indicate Mendelian randomization (MR) as the study's design in the title and/or the abstract if that is a main purpose of the study	2	Abstract: …Uni- (UVMR) and multivariable mendelian randomization (MVMR) were used to evaluate and differentiate the causal effect of childhood and adulthood BMI.
	**INTRODUCTION**				
2	**Background**		Explain the scientific background and rationale for the reported study. What is the exposure? Is a potential causal relationship between exposure and outcome plausible? Justify why MR is a helpful method to address the study question	3-4	Obesity has been identified as a modifiable environmental factor that affect the initiation and progression of AMD, …However, no populational study has been conducted to investigate the association between childhood obesity and incident AMD, and no mendelian randomization study has been performed to assess the potential causal relationship.
3	**Objectives**		State specific objectives clearly, including pre-specified causal hypotheses (if any). State that MR is a method that, under specific assumptions, intends to estimate causal effects	3-4	Uni- and multi-variable mendelian randomization was used to explore potential causal association using objectively-measured childhood and adulthood BMI, free of conventional confounding and reversal causation.
	**METHODS**				
4	**Study design and data sources**		Present key elements of the study design early in the article. Consider including a table listing sources of data for all phases of the study. For each data source contributing to the analysis, describe the following:	Supp. T10	
		a)	Setting: Describe the study design and the underlying population, if possible. Describe the setting, locations, and relevant dates, including periods of recruitment, exposure, follow-up, and data collection, when available.	5-6, Supp. Methods, Supp.T10	A recent meta-analysis using 39,620 European children aged 2-10 years from 26 studies was used to construct instrumental variables (IVs) in this study…
		b)	Participants: Give the eligibility criteria, and the sources and methods of selection of participants. Report the sample size, and whether any power or sample size calculations were carried out prior to the main analysis	5-6, Supp. Methods	A recent meta-analysis using 39,620 European children aged 2-10 years from 26 studies was used to construct instrumental variables (IVs) in this study.
		c)	Describe measurement, quality control and selection of genetic variants	5-6, Supp. Methods	Age- and sex-adjusted BMI Standard Deviation Scores (SDS) were used in the meta-GWAS…
		d)	For each exposure, outcome, and other relevant variables, describe methods of assessment and diagnostic criteria for diseases	5-6	Age- and sex-adjusted BMI Standard Deviation Scores (SDS) were used in the meta-GWAS…
		e)	Provide details of ethics committee approval and participant informed consent, if relevant	NA	
5	**Assumptions**		Explicitly state the three core IV assumptions for the main analysis (relevance, independence and exclusion restriction) as well assumptions for any additional or sensitivity analysis	Supp. Methods	Mendelian randomization uses genetic variants as IVs to estimate causal effect of exposure on outcome, unbiased by residual confounding and reverse causation…
6	**Statistical methods: main analysis**		Describe statistical methods and statistics used		
		a)	Describe how quantitative variables were handled in the analyses (i.e., scale, units, model)	Supp. Methods	Age- and sex-adjusted BMI Standard Deviation Scores (SDS) were used in the meta-GWAS…
		b)	Describe how genetic variants were handled in the analyses and, if applicable, how their weights were selected	NA	
		c)	Describe the MR estimator (e.g. two-stage least squares, Wald ratio) and related statistics. Detail the included covariates and, in case of two-sample MR, whether the same covariate set was used for adjustment in the two samples	Supp. Methods	Inverse-variance weighted (IVW) method was adopted as the main analysis,…
		d)	Explain how missing data were addressed	NA	
		e)	If applicable, indicate how multiple testing was addressed	NA	
7	**Assessment of**		Describe any methods or prior knowledge used to assess the assumptions or	Supp. Methods	Therefore, we further performed three sensitivity

	**assumptions**	justify their validity		analysis to test and account for heterogeneity and pleiotropy…
8	**Sensitivity analyses and additional analyses**	Describe any sensitivity analyses or additional analyses performed (e.g. comparison of effect estimates from different approaches, independent replication, bias analytic techniques, validation of instruments, simulations)	Supp. Methods	The MR-Egger method implicates directional pleiotropy thorough non-zero intercept and provides pleiotropy-corrected causal estimates,…
9	**Software and pre-registration**			
	a)	Name statistical software and package(s), including version and settings used	Supp. Methods	All analysis were performed using R version 4.2.1, and “TwoSampleMR” (ver. 0.5.6)), “MRPRESSO” (ver. 1.0), “Mendelian Randomization” (ver. 0.6.0), and “MVMR” (ver. 0.3) R packages were used.
	b)	State whether the study protocol and details were pre-registered (as well as when and where)	NA	
	**RESULTS**			
10	**Descriptive data**			
	a)	Report the numbers of individuals at each stage of included studies and reasons for exclusion. Consider use of a flow diagram	Fig. 1	
	b)	Report summary statistics for phenotypic exposure(s), outcome(s), and other relevant variables (e.g. means, SDs, proportions)	Supp. T10	
	c)	If the data sources include meta-analyses of previous studies, provide the assessments of heterogeneity across these studies	NA	
	d)	For two-sample MR: i. Provide justification of the similarity of the genetic variant-exposure associations between the exposure and outcome samples ii. Provide information on the number of individuals who overlap between the exposure and outcome studies	Supp. Methods	As the GERA study was conducted in United State of America and 6031 participants of the exposure meta-GWAS was American, the sample overlap is no more than 10% in the primary analysis even if all American participants were overlapped….
11	**Main results**			
	a)	Report the associations between genetic variant and exposure, and between genetic variant and outcome, preferably on an interpretable scale	7-8, Fig. 2	Therefore, IVW was adopted as the primary analysis, and significant causal association between per SD increase in childhood BMI and AMD in our primary analysis (IVW OR=1.51, 95%CI, 1.09, 2.08, p=0.013)…
	b)	Report MR estimates of the relationship between exposure and outcome, and the measures of uncertainty from the MR analysis, on an interpretable scale, such as odds ratio or relative risk per SD difference	7-8, Fig. 2	Therefore, IVW was adopted as the primary analysis, and significant causal association between per SD increase in childhood BMI and AMD in our primary analysis (IVW OR=1.51, 95%CI, 1.09, 2.08, p=0.013)…
	c)	If relevant, consider translating estimates of relative risk into absolute risk for a meaningful time period	NA	
	d)	Consider plots to visualize results (e.g. forest plot, scatterplot of associations between genetic variants and outcome versus between genetic variants and exposure)	Fig. 2	
12	**Assessment of assumptions**			
	a)	Report the assessment of the validity of the assumptions	7-8, Fig. 2	In uni-variable mendelian randomization, MR-Egger Intercept (p=0.544) was insignificant, …
	b)	Report any additional statistics (e.g., assessments of heterogeneity across genetic variants, such as I^2, Q statistic or E-value)	7-8, Fig. 2	Q-statistics for IVW (p=0.350) and MR-Egger (p=0.300) demonstrated that there was no significant heterogeneity.
13	**Sensitivity analyses and additional**			

	**analyses**			
	a)	Report any sensitivity analyses to assess the robustness of the main results to violations of the assumptions	7-8, Fig. 2	Weighted median analysis and MR-PRESSO estimates also showed significant associations, further confirmed robustness of our results….
	b)	Report results from other sensitivity analyses or additional analyses	7-8, Fig. 2	Weighted median analysis and MR-PRESSO estimates also showed significant associations, further confirmed robustness of our results….
	c)	Report any assessment of direction of causal relationship (e.g., bidirectional MR)	7-8, Fig. 2	Furthermore, reverse MR and MR Steiger directionality test confirmed the direction. [Supplemental Table 6]
	d)	When relevant, report and compare with estimates from non-MR analyses	5-6	
	e)	Consider additional plots to visualize results (e.g., leave-one-out analyses)	Fig. 2	
	**DISCUSSION**			
14	**Key results**	Summarize key results with reference to study objectives	8	Mendelian randomization analysis also demonstrated the positive association between childhood BMI and AMD, independent of adulthood BMI.
15	**Limitations**	Discuss limitations of the study, taking into account the validity of the IV assumptions, other sources of potential bias, and imprecision. Discuss both direction and magnitude of any potential bias and any efforts to address them	9	However, our study still has some limitations….
16	**Interpretation**			
	a)	Meaning: Give a cautious overall interpretation of results in the context of their limitations and in comparison with other studies	8-9	This study was the first and largest prospective cohort study and mendelian randomization to demonstrate the positive association between plumper childhood body size and incident AMD….
	b)	Mechanism: Discuss underlying biological mechanisms that could drive a potential causal relationship between the investigated exposure and the outcome, and whether the gene-environment equivalence assumption is reasonable. Use causal language carefully, clarifying that IV estimates may provide causal effects only under certain assumptions	8-9	Photoreceptor cell loss has been observed in AMD, and photoreceptor segment layer thinning emerged decades before RPE+BM thickening and drusen deposits. …
	c)	Clinical relevance: Discuss whether the results have clinical or public policy relevance, and to what extent they inform effect sizes of possible interventions	8-9	…All these results together suggest that obesity management should be implemented in a life course manner.
17	**Generalizability**	Discuss the generalizability of the study results (a) to other populations, (b) across other exposure periods/timings, and (c) across other levels of exposure	9-10	First, the study cohort was largely Caucassians aged 40-70, so generalization into other ethnics and age groups was not applicable.
	**OTHER INFORMATION**			
18	**Funding**	Describe sources of funding and the role of funders in the present study and, if applicable, sources of funding for the databases and original study or studies on which the present study is based	10	This study was supported by grants from the National Natural Science Foundation of China (No. 82271111).
19	**Data and data sharing**	Provide the data used to perform all analyses or report where and how the data can be accessed, and reference these sources in the article. Provide the statistical code needed to reproduce the results in the article, or report whether the code is publicly accessible and if so, where	Supp. Methods, Supp. T10	All analysis were performed using R version 4.2.1, and "TwoSampleMR" (ver. 0.5.6)), "MRPRESSO" (ver. 1.0), "Mendelian Randomization" (ver. 0.6.0), and "MVMR" (ver. 0.3) R packages were used.
20	**Conflicts of Interest**	All authors should declare all potential conflicts of interest	10	All authors report no conflicts of interest.

This checklist is copyrighted by the Equator Network under the Creative Commons Attribution 3.0 Unported (CC BY 3.0) license.

1. Skrivankova VW, Richmond RC, Woolf BAR, Yarmolinsky J, Davies NM, Swanson SA, et al. Strengthening the Reporting of Observational Studies in Epidemiology using Mendelian Randomization (STROBE-MR) Statement. JAMA. 2021;under review.

2. Skrivankova VW, Richmond RC, Woolf BAR, Davies NM, Swanson SA, VanderWeele TJ, et al. Strengthening the Reporting of Observational Studies in

Epidemiology using Mendelian Randomisation (STROBE-MR): Explanation and Elaboration. BMJ. 2021;375:n2233.
